# C1GALT1 expression predicts a favorable prognosis and suppresses malignant phenotypes via TrkA signaling in neuroblastoma

**DOI:** 10.1038/s41389-022-00383-w

**Published:** 2022-02-15

**Authors:** Neng-Yu Lin, Syue-Ting Chen, Hsiu-Ling Chang, Meng-Yao Lu, Yung-Li Yang, Shu-Wei Chou, Dong-Tsamn Lin, Kai-Hsin Lin, Shiann-Tarng Jou, Wen-Ming Hsu, Min-Chuan Huang, Hsiu-Hao Chang

**Affiliations:** 1 Graduate Institute of Anatomy and Cell Biology, National Taiwan University College of Medicine, Taipei, Taiwan; 2 Department of Anatomy, College of Medicine, Chang Gung University, Taoyuan, Taiwan; 3 Department of Neurosurgery, Chang Gung Memorial Hospital, Taoyuan, Taiwan; 4 Department of Pediatrics, National Taiwan University Hospital, National Taiwan University College of Medicine, Taipei, Taiwan; 5 Departments of Laboratory Medicine, National Taiwan University Hospital, National Taiwan University College of Medicine, Taipei, Taiwan; 6 Department of Surgery, National Taiwan University Hospital, National Taiwan University College of Medicine, Taipei, Taiwan

**Keywords:** Paediatric cancer, Embryonal neoplasms, Glycobiology

## Abstract

Neuroblastoma (NB) is a childhood tumor derived from the sympathoadrenal lineage of the neural crest progenitor cells. Core 1 β1,3-galactosyltransferase (C1GALT1) controls the crucial step of GalNAc-type O-glycosylation, and its altered expression affects cancer behaviors. However, the role of C1GALT1 in NB tumors remains unclear. Our data showed that C1GALT1 expression was significantly associated with differentiated tumor histology, correlated with TrkA expression, and predicted good prognosis independently in NB. Downregulation of C1GALT1 promotes malignant behaviors of NB cells in vitro and in vivo. Mechanistic investigation showed that knockdown of C1GALT1 in NB cells increased TrkA pulled down through Vicia villosa agglutinin beads, indicating the modulation of O-glycans on TrkA by C1GALT1, and silencing C1GALT1 suppressed the TrkA expression on the NB cell surface. Overexpression of C1GALT1 increased the protein levels of TrkA and promoted the differentiation of NB cells, whereas knockdown of TrkA inhibited C1GALT1-induced neuronal differentiation. Moreover, the inhibitory effects of migration and invasion in C1GALT1-overexpressing NB cells were blocked by TrkA downregulation. C1GALT1 knockdown enhanced AKT phosphorylation but attenuated ERK phosphorylation, and these properties were consistent in C1GALT1-overexpressing NB cells with TrkA knockdown. Taken together, our data provided the first evidence for the existence of GalNAc-type O-glycans on TrkA and altered O-glycan structures by C1GALT1 can regulate TrkA signaling in NB cells. This study sheds light on the novel prognostic role of C1GALT1 in NB and provides new information of C1GALT1 and TrkA on the pathogenesis of NB.

## Introduction

Neuroblastoma (NB) is a childhood tumor derived from sympathoadrenal lineage of the neural crest progenitor cells. It develops in the neurons of sympathetic nervous system, and in the medulla of adrenal gland. It occurs with an incidence of 8.0 per million per year [1], and 96% of cases occur before the age of 10 years [2]. NB represents the most common extracranial solid tumor in children as well as the most frequently diagnosed malignancy during infancy [3]. The clinical presentation of NB can be categorized into three distinct patterns: (i) life-threatening progression and metastasis; (ii) maturation to ganglioneuroblastoma (GNB) or ganglioneuroma (GN); and (iii) spontaneous regression [4]. So NB is quite a heterogeneous tumor and presents a broad clinical and biologic spectrum ranging from highly undifferentiated tumors with very poor outcomes to the most differentiated benign GN or NB with spontaneous regression and hence favorable prognosis [3]. Although NB represents 5–8% of all malignant childhood diseases, it is disproportionately responsible for up to 10–15% of cancer deaths in children [5].

NB patients can be classified into different risk groups for clinical treatment based on relevant biologic prognostic factors [6, 7]. Among these factors, MYCN proto-oncogene amplification, which is detected in about 20–30% of NB, is considered as the most reliable genomic hallmark of aggressive tumor behavior and treatment failure [3, 6, 8, 9]. MYCN expression is well known to be suppressed in differentiating NB cells by TrkA activation with its ligand nerve growth factor (NGF) [10]. TrkA is one of the members of the Trk family which are receptor tyrosine kinase (RTKs) [10]. The Trk family receptors are initially synthesized as precursor proteins; the post-translational glycosylation of the extracellular domains of these precursors is required to localize TrkA to the cell surface, where it can trigger the Ras/MAP/ERK kinase cascade for neurotrophin-promoted differentiation of neurons [11–14].

Glycosylation is one of the most common posttranslational modifications of proteins in mammalian cells and alterations in glycosylation regulate the development and progression of cancer [15]. Altered glycan structures on cell surfaces are prominent features of cancer cells and play important roles to affect cell behaviors. GalNAc-type O-glycosylation is the most common type of O-glycosylation, and it modulates diverse functions of membrane-bound and secreted proteins [16]. During GalNAc-type O-glycosylation, the GALNT family enzymes transfer N-acetylgalactosamine (GalNAc) from UDP-GalNAc to serine (Ser) or threonine (Thr) residues to form the GalNAc-O-Ser/Thr structure, also known as Tn antigen [17]. Subsequently, core 1 β1,3-galactosyltransferase (C1GALT1), an exclusive T-synthase in mammalian cells, catalyzes the transfer of galactose (Gal) from UDP-Gal to the Tn antigen, forming Gal-GalNAc-O-Ser/Thr structure, known as T antigen or core 1 structure. The core 1 structure is a basis for further complex O-glycan formation, such as core 2 structure [18]. ClGALT1 plays critical roles in many biological functions, and its deletion results in developmental defects, spontaneous colitis and thrombocytopenia in mice [18–21]. Moreover, aberrant expression of truncated O-glycans is a characteristic mark observed on the surface of tumor cells, and is associated with an adverse outcome and poor prognosis in patients with several malignant diseases [22]. Previous studies showed that C1GALT1 is overexpressed in hepatocellular carcinoma, colorectal cancer, breast cancer, head, and neck squamous cell carcinoma, and gastric cancer [23–28]; and increased C1GALT1 expression correlates with higher histological grade, or advanced tumor stage and poor survivals in the above cancers. At the molecular level, manipulating the expression of C1GALT1 has been demonstrated to regulate O-glycosylation of MET in hepatocellular carcinoma, FGFR2 in colorectal cancer, Mucin 1 in breast cancer, EGFR in head and neck squamous cell carcinoma cells, and EPHA2 in gastric cancer, respectively, and affects cancer malignant behaviors [23, 24, 26–28]. However, unlike the above adult tumors, NB is the most common extracranial solid tumor in children, and rare in adults [29]. The pathophysiologic mechanism of C1GALT1 in NB tumors remains largely unclear.

The present study is the first report to show that high C1GALT1 expression correlates with NB tumor differentiation status and predicts better survival outcomes of patients with NB. The chi-square analysis also indicated positive correlations between the expression of TrkA and C1GALT1 in NB tumors. In addition, downregulation of C1GALT1 promotes malignant behaviors of NB cells in vitro and in vivo by altering TrkA glycosylation and its downstream signaling pathways.

## Materials and methods

### Patients and treatment

From December 1990 to December 2020, 134 histologically proved NB patients with complete follow-up were included in this study. All diagnoses of tumors were confirmed by histologic assessment of a specimen obtained from the primary or metastatic tumor at surgery. Based on the criteria of the International NB Pathology Classification [30], the differentiating status of the tumor histology was categorized into undifferentiated NB (UNB, Schwannian stroma poor), differentiating NB (DNB, Schwannian stroma poor, including poorly differentiated subtype), and ganglioneuroblastoma, intermixed (GNB, Schwannian stroma-rich) according to the percentage and degree of differentiation of the NB cells. The nodular type GNB was classified into either undifferentiated or differentiating NB according to the morphology of their NB nodules, since the prognosis of this type of tumors depends mainly on their NB nodules [31]. The distinction of tumor staging was based on the International NB Staging System (INSS) [32]. MYCN status of the tumor tissue was evaluated by chromogenic in situ hybridization analysis of formalin-fixed paraffin-embedded tissues or fresh tumor single cells [33]. The treatment of these patients has been described in previous studies [34–36]. The clinical parameters, including age at diagnosis, sex, primary tumor sites, clinical stages, differentiating status of tumor histology, and MYCN amplification status were collected for prognostic and survival analysis. Written consent was obtained from the patients. This study was approved by the Institutional Review Board of the National Taiwan University Hospital (IRB No: 201812005RIND).

### Immunohistochemistry

One hundred eighty-four tumor specimens collected before chemotherapy were fixed in formalin and embedded in paraffin. The expression of C1GALT1 was evaluated by using an avidin-biotin complex immunoperoxidase staining technique as described previously [37]. A monoclonal anti-C1GALT1 antibody (Santa Cruz) was used and signals were detected with Super Sensitive Link-Label immunohisto-chemistry Detection System (BioGenex). The specific staining was visualized with 3,3-diaminobenzidine liquid substrate system (Sigma) and counterstained with hematoxylin (Sigma).

### Cell lines and cell culture

Human NB cells, SK-N-BE, SKN-SH, and GI-LI-N, were kindly provided by Dr. Yung-Feng Liao, Institute of Cellular and Organismic Biology, Academia Sinica, Taipei, Taiwan. These cell lines were cultured with Dulbecco’s modified Eagle’s medium (DMEM; Invitrogen) containing 10% FBS (Invitrogen), 100 IU/mL penicillin, and 100 μg/mL streptomycin (Invitrogen) in a humidified tissue culture incubator at 37 °C and 5% CO_2_ atmosphere. All these cell lines were authenticated by short tandem repeat profiling and negativity of mycoplasma contamination was routinely checked through PCR every week. For stable knockdown and overexpression of C1GALT1 in NB cells, sh-C1GALT1/pLKO.1-puro and sh-control/pLKO.1-puro (RNAi Core, Academia Sinica) were used in the lentivirus-based infection system and stable pooled clones were selected and maintained with 1 μg/mL puromycin (Sigma). The following target sequences were used: shC1GALT1#1 (5′ CCCAGCCTAATGTTCTTCATA 3′); shC1GALT1#2 (5′ GCTCTGATCTTGCAGTTTCTT 3′).

### Quantitative real time-PCR

Total RNA was purified using NucleoZOL reagent (MACHEREY-NAGEL, Germany) and 1 μg RNAs were prepared for cDNA synthesis by MMLV Reverse Transcription kit (Protech Technology Enterprise, Taiwan). Gene expression was quantified by SYBR Green qPCR. The following primer pairs were used: human C1GALT1: 5′-GAAAGTTTGCCTGGGTCCTCT TGG GAGAAAAGGTTGACACC-3′ and 5′-CTGGGATGCGGAGAGCGCTTTGACGTGTTTGGCCTTT-3; human TrKA: 5′-AGAACACGCGTATTTACCTGC CCTGCTGGCTTGGCTGATACT-3′ and 5′-ACCATGAGTCTAACATCTCCCAC ACGGAGACCACTCTTCACGA-3′; human β-actin, 5′-CGTGCGTGACATTAAGGAGA-3′ and 5′-GAAGGAAGGCTGGAAGAGTG-3′; β-actin was used to normalize for the amounts of loaded cDNA.

### Western blot analysis and lectin pull-down assay

Cell lysates were extracted in lysis buffer (Thermo Fisher Scientific). Proteins were separated on a 10% SDS-PAGE and transferred onto PVDF membranes. The membranes were blocked in TBST containing 5% bovine serum albumin (Bio-Rad) and then incubated with monoclonal antibodies for C1GALT1 (Santa Cruz Biotechnology), GAPDH (Santa Cruz Biotechnology), TrkA (LSBio), AKT, and pAKT (Ser536) (Cell Signaling Technology) and polyclonal antibodies for ERK1/2 (phospho Thr202/Tyr204, phosphor Thr185/Tyr187) antibody and ERK1/2 (GeneTex) and biotinylated *Vicia villosa* lectins (VVA or VVL) (Vector Laboratories). After incubation with horseradish peroxidase (HRP)-conjugated secondary antibodies and HRP-conjugated streptavidin, protein bands were detected with ECL reagents (GE Healthcare Life Sciences). For lectin pull-down assay, 500 μg of total proteins from cell lysates with or without 0.4 U/mL neuraminidase (Sigma-Aldrich) treatment were incubated with VVA, Griffonia simplicifolia lectin I (GSL-I), and Peanut agglutinin (PNA) lectin-conjugated beads (Vector Laboratories). The beads were washed and boiled to pull down proteins. The samples can be separated by SDS-Polyacrylamide Gel Electrophoresis for western blot analysis.

### Immunofluorescence microscopy

Cells were cultured in chamber slides (SPL Life Sciences), fixed with 4% PFA, permeabilized with 0.25% Triton X100, blocked in PBS containing 2% bovine serum albumin (Bio-Rad) and applied with primary antibodies to TrkA (LSBio), Lamp1 (GeneTex). Alexa Fluor antibodies (Life Technologies) were used as secondary antibodies. Isotype control antibodies were used as controls. Cell nuclei were visualized with DAPI (Santa Cruz Biotechnology). Images were captured using a Nexcope NE950 fluorescent microscope (Ningbo Yongxin Optics Co., United States).

### Colony formation assay

Each group of transfected cells were seeded in six-well plates (500 cells per well) and cultured as indicated. At day 14, cells were washed with PBS, fixed with 4% formaldehyde, and stained with 0.1% crystal violet.

### Transwell migration and Matrigel invasion assays

Cell migration and invasion assays were evaluated using transwell (Corning, NY, USA) or Matrigel-coated (BD Biosciences, CA, USA) transwell chamber, respectively. Each transwell chamber contained a membrane filter with pore size 8 μm. Each group of transfected NB cells (1 × 10^5^) were seeded into the transwell or Matrigel-coated transwell chamber contained with 0.25 mL serum-free DMEM. After incubating for 48 h, the cells were fixed and stained with 0.5% (w/v) crystal violet (Sigma) containing 20% (v/v) methanol. The migrated and invaded cells from three random fields were counted under a microscope.

### Animals

All animal experiments were carried out in accordance with a study protocol and guidelines approved by the National Taiwan University College of Medicine and College of Public Health Institutional Animal Care and Use Committee (IACUC). Female NOD-SCID mice aged 6 weeks were purchased from the National Laboratory Animal Center, Taipei, Taiwan. For experimental metastasis analysis, 5 × 10^6^ SK-N-BE and GI-LI-N cells (mock and shC1GALT1) were intracardially injected into the ventricles. Animals were sacrificed at day 40 for evaluation of lung metastasis.

### Statistical analysis

The statistical analysis was carried out with SPSS 17.0 for Windows software. Associations between pairs of categorical variables were assessed with Pearson’s chi-square test. Survival probabilities in various subgroups were estimated using the Kaplan–Meier method, and analyzed by log-rank tests. The influence of each variable on survival was assessed by the univariate and multivariate Cox proportional hazard model. The significance of the variations between the data resulting from different experiments was analyzed by Student’s *t*-test. All statistical tests were two-sided, and those with a *p* value < 0.05 were considered to be statistically significant.

## Results

### High expression of C1GALT1 protein correlates with the differentiation status of NB tumors and predicts better survival outcomes

**Fig. 1:**
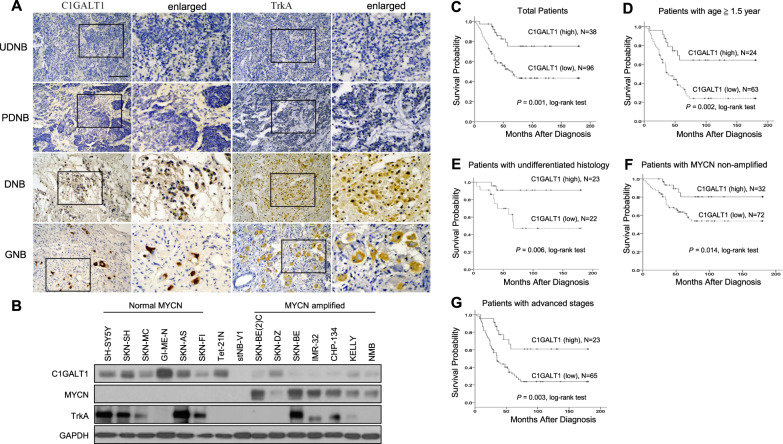
C1GALT1 expression predicts better prognosis for patients with NB. **A** Representative images of IHC staining for C1GALT1 protein in NB tumors with indicated histological types. Brown colors represent positively stained cells. Tumors with more mature differentiation (GNB or DNB) exhibit higher expression levels of C1GALT1 protein in general. Inversely, UNB and NB tumors exhibit lower levels of C1GALT1 expression. Scale bars, 100 μm. High magnified images of the area are marked by the rectangle. **B** Immunoblotting analysis of C1GALT1, MYCN, and TrkA expression in various NB cell lines with MYCN amplification or non- amplification, using GAPDH as internal control. **C** Kaplan–Meier overall survival analysis according to the expression of C1GALT1 in 134 patients with NB (*p* = 0.001, log-rank test). **D**–**G** Kaplan–Meier survival analysis according to the expression of C1GALT1 in patients with age ≧1.5 years (*p* = 0.002, log-rank test), undifferentiated histology (*p* = 0.006, log-rank test), MYCN non-amplified (*p* = 0.014, log-rank test), advanced-stage disease (*p* = 0.003, log-rank test), respectively.

We examined the C1GALT1 protein expression in 134 NB tumors by immunohistochemical (IHC) staining. Positive C1GALT1 staining was seen in ganglion cells, and localized in the cytoplasm in NB. No schwannian stromal cells showed positive staining of C1GALT1. The immunoreactivity of C1GALT1 was classified into four categories: “−” (no expression, no stained cells or only isolated single stained cells seen), “1+” (weak expression, around 10–35% of cells stained), “2+” (moderate expression, around 35–70% of cells stained) and “3+” (strong expression, more than 70% of cells stained). We found that tumors with more mature differentiation histology (GNB or differentiating NB (DNB)) exhibit higher expression levels of C1GALT1 protein (Fig. 1A). Similar correlations were observed in the Western blot analysis of human NB cell lines, showing the protein levels of C1GALT1 in MYCN-non-amplified NB cell lines are mostly higher than that in MYCN-amplified NB cell lines (Fig. 1B). In addition, high expression (immunoreactivity 2+ to 3+) of C1GALT1 protein could be detected in 38 of the 134 NB tumors (28.4%). The majority of NB tumors with high C1GALT1 protein expression were of differentiated histology, differentiated NB cells or ganglion cells. The intensity and percentage of positive C1GALT1 immunostaining correlated strongly with the differentiation of histology. Kaplan–Meier analysis showed that patients with C1GALT1 high expression had a better overall survival rate than those with C1GALT1 low expression (immunoreactivity “−” or “1+”) (*p* < 0.001, log-rank test) (Fig. 1C).

### High expression of C1GALT1 is an independent favorable prognostic marker of NB

The relationship between C1GALT1 expression and clinicopathologic and biologic variables of NB tumors was summarized in Table 1. Thirty of 134 (22.4%) NB tumors were recognized as MYCN amplification. Tumors were assigned into C1GALT1 low expression (“−” or “1+” in immunoreactivity) and high expression (“2+” or “3+” in immunoreactivity) in the statistical analysis. C1GALT1 high expression was significantly associated with differentiated tumor histology (*p* < 0.001, Table 1). Furthermore, univariate analysis showed that, in addition to C1GALT1 low expression, patient age at diagnosis >1.5 years, advanced clinical stage (INSS stage 3 and 4), MYCN amplification, and undifferentiated histology strongly correlated with poor survival (Table 2). Multivariate analysis revealed that the clinical stage, MYCN amplification, undifferentiated histology, and C1GALT1 low expression remained independent prognostic factors for poor survival (Table 2). To further evaluate the significance of C1GALT1 expression in prognostic discrimination, the impact of C1GALT1 expression on survival probability was analyzed against age of patients, differentiation status of tumor, MYCN amplification status, and clinical stages. Results revealed that C1GALT1 high expression in NB tumors constantly predicted better survival outcomes for patients with age >1.5 years (Fig. 1D, *p* = 0.002, log-rank test), undifferentiated tumor histology (Fig. 1E, *p* = 0.006, log-rank test), MYCN non-amplified (Fig. 1F, *p* = 0.014, log-rank test), and advanced stages (Fig. 1G, *p* = 0.003, log-rank test). In addition, we used SEQC-498 and Asgharzadeh-249 datasets from R2: “Genomics Analysis and Visualization Platform“ as independent cohorts to validate our findings of C1GALT1 expression in NB tumors. The analytical results showed that *C1GALT1* high expression in NB predicts better survival outcomes compared with *C1GALT1* low expression (*p* = 1.5e−03 and *p* = 0.011, respectively). Furthermore, *C1GALT1* high expression in NB tumors correlated with good clinical prognostic factors, such as non-high risk (*p* = 0.033) and MYCN non-amplified (*p* = 1.45e−10) status (Supplementary Fig. 1). These are compatible with the findings of C1GALT1 expression in our cohort. All these observations suggest that C1GALT1 expression is an independent prognostic factor for better survival outcomes in NB patients, and could provide complimentary prognostic information in addition to age of patients, differentiation status of tumor, MYCN amplification status, and clinical stages.

**Table 1:** C1GALT1 expression and clinicopathologic and biologic characteristics of NB.

	Cases	C1GALT1 high expression (%)	*p* value
Sex			
Male	84	18 (21.4)	0.029*
Female	50	20 (40.0)	
Primary tumor site			
Adrenal	89	24 (27.0)	0.686
Extra-adrenal	45	14 (31.1)	
Age at diagnosis			
≤1.5 year	47	14 (29.8)	0.842
>1.5 year	87	24 (27.6)	
Clinical stage			
1, 2, 4 S	46	15 (32.6)	0.429
3, 4	88	23 (26.1)	
Tumor histology			
Undifferentiated NB^a^	89	15 (16.9)	<0.001*
Differentiated NB^b^	45	23 (51.1)	
MYCN			
Amplified	30	6 (20.0)	0.358
Non-amplified	104	32 (30.8)	

*p < 0.05.

^a^Including undifferentiated NB and poorly differentiated NB.

^b^Including differentiating NB and ganglioneuroblastoma.

**Table 2:** Clinicopathologic and biologic factors affecting survival rate in NB patients.

Variable	Univariate analysis			Multivariate analysis		
	RR	95% CI	*p* value	RR	95% CI	*p* value
Age at diagnosis: >1.5 year versus ≤1.5 year	4.415	2.262–4.415	<0.001*	1.961	0.765–5.022	0.161
Clinical stage: advanced (3, 4) versus early (1, 2, 4 S)	12.978	4.719–35.689	<0.001*	5.167	1.537–17.374	0.008*
MYCN: amplified versus non-amplified	3.867	2.413–6.199	<0.001*	2.169	1.243–3.783	0.006*
C1GALT1 protein expression: low versus high expression	3.142	1.488–6.634	0.003*	2.329	1.056–5.136	0.036*
Histology: undifferentiated versus differentiated	3.119	1.642–5.923	0.001*	2.071	1.027–4.174	0.042*

*p < 0.05.

### C1GALT1 expression correlates with TrkA expression in NB tumors

Since MYCN expression is well known to be suppressed in differentiating NB cells by TrkA activation with its ligand, NGF, and high TrkA expression in primary NB was associated with favorable clinical features and inversely associated with MYCN amplification [10, 38]. Furthermore, NGF/TrkA signaling was noted to affect NB differentiation or regression depending on the particular microenvironment [3]. These observations of TrkA activation or expression on NB are compatible with our results for the effects of C1GALT1 expression on NB. We thus investigated if C1GALT1 expression correlates with and affects TrkA expression in NB tumors. We examined the TrkA protein expression in 46 NB tumors by IHC staining and results revealed that TrkA expression significantly (*p* = 0.001) and positively correlated with C1GALT1 expression in NB (Supplementary Table 1).

### Downregulation of C1GALT1 promotes malignant behaviors of NB cells in vitro and in vivo

**Fig. 2:**
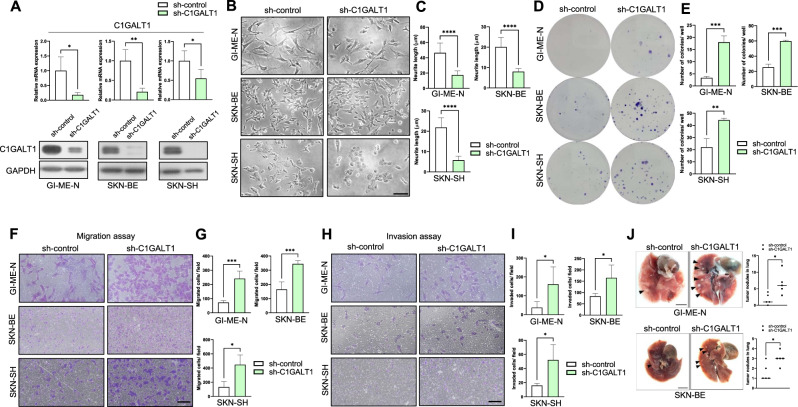
Downregulation of C1GALT1 promoted the malignant phenotypes of NB cells in vitro and in vivo. **A** qRT-PCR and Western blot analysis of C1GALT1 expression in GI-ME-N, SK-N-BE, and SK-N-SH cells infected with shC1GALT1 or negative control lentivirus. **B** Representative images showing neurite outgrowth of NB cells transfected with control shRNA or C1GALT1 shRNA lentivirus for 3 d were collected under a phase-contrast microscope. Scale bar indicates 100 μm. Arrowheads indicate neurite outgrowth. **C** Bars represent the mean of three independent experiments. **D** Representative pictures showed the ability of colony formation in NB cells infected with shC1GALT1 or negative control lentivirus. **E** Bars represent the mean number of colonies (*n* = 3) (magnification, ×100) **p < 0.01, ***p < 0.001. **F** Representative pictures showed transwell migration assay in NB cells infected with shC1GALT1 or negative control lentivirus. G Bars represent the mean number of migrated cells (*n* = 3). Scale bar indicates 100 μm. **H** Representative pictures showed transwell invasion assay in NB cells infected with shC1GALT1 or negative control lentivirus. **I** Bars represent the mean number of invaded cells (*n* = 3). Scale bar indicates 100 μm. **J** Representative macroscopic images and quantification showed the metastatic lung nodules of mice intracardially injected with NB cells infected with shC1GALT1 or negative control lentivirus. Arrowheads indicate the metastatic nodules. Scale bar indicates 5 mm. *p < 0.05, **p < 0.01, ***p < 0.001, ****p < 0.0001.

C1GALT1 was differentially expressed in various kinds of NB cell lines, most notably in MYCN-non-amplified cells (Fig. 1B). To further determine the function of C1GALT1 in NB malignant behaviors, cell proliferation, colony formation, migration, and invasion were analyzed. First, real-time RT-PCR and Western blot analysis confirmed that the mRNA and protein levels of C1GALT1 were significantly downregulated in stable NB cell lines, GI-ME-N, SK-N-BE, and SK-N-AS, on day 20 post transfection (Fig. 2A). The representative bright-field microscopy images and the analysis indicated that C1GALT1 knockdown reduced neurite outgrowth in NB cells (Fig. 2B, C). Supportively, the real-time RT-PCR analysis also showed that C1GALT1 knockdown inhibited mRNA expression of differentiation markers in NB cells (Supplementary Fig. 2). In addition, the colony formation analysis showed that knockdown of C1GALT1 significantly increased colony formation compared with the mock transfectants (Fig. 2D, E). In transwell migration and Matrigel invasion assays, C1GALT1 knockdown increased NB cell migration and invasion (Fig. 2F–I). These studies suggested that inhibition of C1GALT1 enhances malignant phenotypes in NB cells. We next examined whether this effect also occurs in vivo. GI-ME-N and SK-N-BE cells stably transfected with shC1GALT1 or control lentivirus were intracardially injected into 6-week-old NOD-SCID mice. Animals were sacrificed at day 40 for evaluation of lung metastasis. The results showed that silencing C1GALT1 increased metastatic nodules in the lungs of NOD-SCID mice received GI-ME-N and SK-N-BE transfectants (Fig. 2J).

### C1GALT1-mediated O-glycans regulate cell surface expression and signaling of TrkA in NB cells

**Fig. 3:**
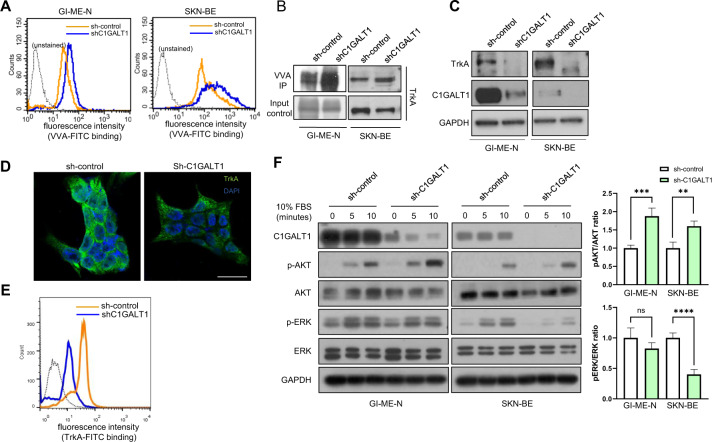
Downregulation of C1GALT1-mediated O-glycan change regulates the protein expression and localization of TrkA and its downstream signaling. **A**–**B** C1GALT1 could modify the Tn antigen expression on TrkA in NB cells. **A** Representative histogram plots from three independent experiments are shown for flow cytometry with FITC-VVA lectin in GI-ME-N and SK-N-BE cells infected with shC1GALT1 or negative control lentivirus. **B** Total lysates were pulled down (PD) using VVA agarose beads and then immunoblotted with a TrkA antibody. **C** Expression of TrkA, C1GALT1, and GAPDH in whole lysates was shown by Western blotting. **D**–**E** Inhibition of C1GALT1 could regulate the localization of TrkA. **D** Representative immunofluorescence images taken from confocal microscope show the localization of TrkA (green) in cells. DAPI (blue) was used to stain the nucleus. Scale bar indicates 50 μm. **E** Representative histogram plots showed TrkA expression levels at the cell surface measured by flow cytometry. **F** C1GALT1 knockdown decreased phosphorylation of ERK, but increased phosphorylation of AKT in GI-ME-N and SK-N-BE cells. Serum-starved cells were reintroduced with 10% FBS for different times as indicated. Western blots show the expression of AKT, p-AKT, ERK, p-ERK, C1GALT1, and GAPDH (*n* = 3). The bar diagrams of the ratios of p-AKT/total AKT and p-ERK/total ERK were shown in the right panel. **p < 0.01, ***p < 0.001, ****p < 0.0001.

We have identified that multiple RTKs, such as EGFR, FGFR2, EPHA2, and MET, are key targets for C1GALT1 and their appropriate O-glycosylation is essential for cancer progression and metastasis [24, 26–28]. Surprisingly, the effects of O-glycosylation on the TrkA receptor, the crucial RTKs for the differentiation of sympathetic and sensory neurons, have not yet been fully investigated in NB. First, we investigated whether manipulating C1GALT1 expression affects O-glycosylation in NB cells. Flow cytometry revealed that knockdown of C1GALT1 enhanced *Vicia villosa* agglutinin (VVA) lectin binding to cell surfaces in GI-LN-M and SK-N-BE cells (Fig. 3A). To further confirm the presence of C1GALT1-mediated O-glycosylation on TrkA, C1GALT1 knockdown or overexpressing NB cells were analyzed using VVA, GSL-I, and PNA lectin pull-down and TrkA immunoblotting assays, respectively. The results showed that knockdown of C1GALT1 in NB cells increased TrkA pulled down through VVA and GSL-I lectin beads. On the other hand, overexpression of C1GALT1 in NB cells increased TrkA pulled down through PNA lectin beads. The effect of VVA and GSL-I lectin binding is more obvious after enzymatic desialylation. These results indicated that modifying the C1GALT1 expression indeed alters O-glycosylation of TrkA in NB cells. (Fig. 3B, Supplementary Fig. 3). However, the effects of C1GALT1-mediated O-glycosylation on TrkA are unclear. We first analyzed the levels of TrkA mRNA in mock and shC1GALT1 transfectants. The real-time RT-PCR analysis showed no significant change in the mRNA levels of *TrkA* in shC1GALT1 transfected NB cells (Supplementary Fig. 4). We further performed Western blotting and immunofluorescence staining to detect the expression and examine the localization of TrkA protein in C1GALT1 knockdown cells, respectively. The Western blotting results showed that TrkA protein expression level was decreased in both C1GALT1 knockdown GI-ME-N and SK-N-BE cells (Fig. 3C). The representative fluorescent images taken using confocal microscopy demonstrated that TrkA was predominantly observed on intracellular vesicles in C1GALT1 knockdown transfectants (Fig. 3D). Moreover, flow cytometry consistently provided evidence that silencing C1GALT1 suppressed the localization of TrkA on the surface in SKN-BE cells (Fig. 3E). Because TrkA activates several signaling pathways responsible for the differentiation and proliferation of NB cells, we, therefore, hypothesized that the C1GALT1-mediated alteration in TrkA O-glycans may affect its downstream signaling. The immunoblotting results and the relative quantification of the ratios of p-AKT/total AKT and p-ERK/total ERK showed that the level of phosphorylated AKT was significantly increased in C1GALT1 knockdown cells, but attenuated the level of phosphorylated ERK, suggesting that inhibition of C1GALT1 is involved in sustained AKT signaling downstream of TrkA (Fig. 3F).

### TrkA signaling pathways are involved in the phenotypic changes mediated by C1GALT1

**Fig. 4:**
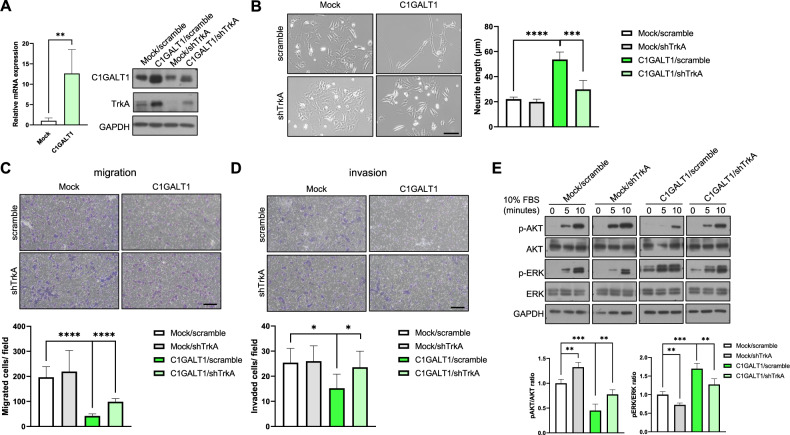
Overexpression of C1GALT1 mediates antitumor effects on NB cells through TrkA. **A** qRT-PCR analysis showed the mRNA expression levels of C1GALT1 in mock and C1GALT1-overexpressing SK-N-SH cells. Western blot analysis showed the protein expression levels of C1GALT1, TrkA and GAPDH in SK-N-SH transfectants infected with shTrkA or negative control lentivirus. **B**–**D** C1GALT1 overexpression-mediated antitumor effects were attenuated by silencing TrkA. **B** Representative images showed neurite outgrowth of SK-N-SH transfectants infected with shTrkA or negative control lentivirus for 3 d photographed under a phase-contrast microscope. Scale bar indicates 100 μm. Arrowheads indicate neurite outgrowth. Bars represent the mean of three independent experiments. **C** Representative pictures showed the ability of transwell migration in SK-N-SH transfectants infected with shTrkA or negative control lentivirus. Bars (lower panel) represent the mean number of migrated cells (*n* = 3, scale bar indicates 100 μm). **D** Representative pictures showed Matrigel invasion assay in SK-N-SH transfectants infected with shTrkA or negative control lentivirus. Bars (lower panel) represent the mean number of invaded cells (*n* = 3, scale bar indicates 100 μm). **E** C1GALT1 overexpression-mediated changes in AKT and ERK phosphorylation were reversed by silencing TrkA. Serum-starved cells were reintroduced with 10% FBS for different times as indicated. Western blots show the expression of AKT, p-AKT, ERK, p-ERK, C1GALT1, and GAPDH (*n* = 3). The bar diagrams of the ratios of p-AKT/total AKT and p-ERK/total ERK were shown in the right panel. *p < 0.05, **p < 0.01, ***p < 0.001, ****p < 0.0001.

We next aimed to determine whether upregulation of C1GALT1 may contribute to the antitumor phenotypes of NB cells and promote neurite outgrowth through TrkA pathway. Overexpression of C1GALT1 increased the protein levels of TrkA (Fig. 4A) and promoted the differentiation of NB cells with increased the growth of neurites, whereas knockdown of TrkA inhibited C1GALT1-induced neuronal differentiation (Fig. 4B). Moreover, the inhibitory effects of migration and invasion in C1GALT1-overexpressing NB cells were blocked by shRNA-mediated TrkA downregulation (Fig. 4C, D). Since the activation of ERK and AKT signaling are the major response of NGF/TrkA for neuronal cell differentiation and proliferation, respectively [39], we next analyzed the phosphorylation levels of ERK and AKT in the C1GALT1-overexpressing and TrkA knockdown NB cells. Similar as C1GALT1 knockdown, the bar diagram of the ratios of p-AKT/total AKT and p-ERK/total ERK indicated that TrkA knockdown also decreased C1GALT1-induced phosphorylation of ERK, but reversely increased phosphorylation of AKT in SK-N-SH cells (Fig. 4E). Collectively, these results indicated that TrkA signaling pathways are at least partially involved in C1GALT1-mediated malignant phenotypes of NB cells.

## Discussion

In this study, the analysis of tumor samples from a separate, larger cohort of NB patients demonstrated that C1GALT1 was highly expressed in ganglion cells and well-differentiated NB; and that C1GALT1 high expression correlated with clinicopathologic features including age, disease stage, histologic classification, and the expression of TrkA. C1GALT1 high expression is an independent prognostic factor for better survival outcomes in NB patients, and could provide complementary prognostic information in addition to age of patients, differentiation status of tumor, MYCN amplification status, and clinical stages. Knockdown of C1GALT1 promoted malignant properties of NB cells in vitro and enhanced the pulmonary metastases of NB in vivo. Moreover, our results demonstrated that O-glycosylation is a critical regulatory factor for TrkA stability and signaling. Silencing of C1GALT1 truncated O-glycans on TrkA, inhibited the presence of TrkA on the plasma membrane, and enhanced migration and invasion in NB cells, whereas overexpression of C1GALT1 promoted cell differentiation and suppressed the migration and invasion of NB cells. Furthermore, we also found that the effect of C1GALT1 knockdown on cell malignant phenotypes was reproduced by silencing TrkA in C1GALT1-overexpressing NB cells. These results suggest that C1GALT1-mediated changes in TrkA O-glycosylation partly regulate the malignant behaviors of NB cells. This is the first study to demonstrate the effect of O-glycosylation in TrkA signaling in NB. Our findings highlight the prognostic role of C1GALT1 in NB, and provide novel information of C1GALT1 and TrkA on the pathogenesis of NB, which could further help in developing new therapeutic targets or strategies for NB in the future.

RTKs mediate intracellular signaling networks that are involved in the progression of tumorigenesis by promoting cell proliferation, differentiation, survival and cell migration [40]. NB is a clinically heterogeneous pediatric cancer of the sympathetic nervous system that originates from neural crest cells. At present, current evidence suggests that the RTK family of neurotrophin receptors play a critical role in NB development [41]. Particularly, NB tumors expressing TrkA are prone to spontaneous regression or differentiation and strongly correlated with low stage in the clinical relevance [41, 42]. Interestingly, the results of our study revealed that TrkA expression was positively correlated with C1GALT1 (*p* = 0.001, Supplementary Table 1) in NB tumors, and C1GALT1 high expression was significantly associated with differentiated tumor histology (Table 1). The Kaplan–Meier analysis in our NB cohort showed that patients with high expression of C1GALT1 had a better survival probability compared with those with low expression of C1GALT1. The multivariate Cox regression analysis in our NB cohort also indicated that C1GALT1 high expression was a significant independent predictor of better survival. Therefore, our clinical data strongly suggested that C1GALT1 could be a potential good prognostic marker for NB patients and the underlying mechanism by which C1GALT1 affects phenotypic changes of NB may be through TrkA signaling.

Several studies have demonstrated that N-glycosylation is of crucial importance in ligand binding, kinase activity, and the determination of the proper conformation and translocation of the RTKs [14, 43–45]. In addition, our previous studies also identified the stimulatory effect of C1GALT1-mediated O-glycosylation on RTK activity such as EPHA2, integrin, and MET in several adult cancers and C1GALT1 high expression was associated with poor survival of patients in these studies [24, 26–28, 46]. Interestingly, high expression of C1GALT1 in tumor tissues predicts a better prognosis in NB in our current study. Another study showed loss of C1GALT1 in pancreatic cancer is correlated with poor prognosis and metastasis [47]. These observations indicate that the association of C1GALT1 expression and survival in cancer patients is highly dependent on tumor types. In some tumors, C1GALT1 plays a tumor-promoting role, while in others C1GALT1 is tumor-suppressive. In addition, the role of C1GALT1-mediated O-glycosylation in TrkA has not yet been investigated in the literature and we, therefore, focused on this issue. Our data showed that silencing C1GALT1 increased VVA and GSL-I lectin binding to TrkA, indicating enriched short O-glycans (Tn antigens) on TrkA. C1GALT1 knockdown decreased the protein expression level of TrkA and its presence on the plasma membrane. Our data provided the first experimental evidence for the existence of GalNAc-type O-glycans on TrkA and altered O-glycan structures can regulate TrkA signaling. Supportively, the NetOglyc server developed by Henrik Clausen’s team predicts several consecutive O-glycosylation sites (366-368) in TrkA [48]. Moreover, the inhibitory effects of C1GALT1 overexpression on cell migration, invasion, and neurite outgrowth were countervailed by TrkA knockdown. These results suggest that silencing C1GALT1 alters O-glycans on TrkA to decrease its protein level and consequently promotes malignant phenotypes in NB cells.

After the binding of neurotrophic factors, activated Trk receptors will participate in various intracellular signaling pathways, such as extracellular signal-regulated kinase (ERK), phosphatidylinositol 3-kinase (PI3K), Protein kinase B (AKT) and phospholipase Cγ (PLC-γ) mediated signaling pathways, to promote cell differentiation, growth, and survival, as well as to change synaptic plasticity and cell motility [13, 14, 49]. Indeed, our Western blotting data showed that C1GALT1 knockdown decreased phospho-ERK levels, but increased phospho-AKT levels in NB cells, whereas overexpression of C1GALT1 elevated phospho-ERK levels, but decreased phospho-AKT levels. Moreover, the effects of C1GALT1 overexpression on the regulation of ERK and AKT pathways were abrogated by silencing TrkA with shRNA. The signaling patterns are consistent with in vitro phenotypes, suggesting that C1GALT1-mediated O-glycosylation can determine TrkA signaling for neuronal differentiation. These findings support the regulatory effect of O-glycans on RTK functions and imply regulating O-glycosylation on TrkA as a potent therapeutic strategy for NB.

Taken together, our study showed that C1GALT1 high expression is an independent prognostic factor and predicts better survival outcomes for NB patients, complementary to age of patients, differentiation status of tumor, MYCN status, and clinical stages. Silencing C1GALT1 decreases TrkA expression and promotes malignant phenotypes in NB in vitro and in vivo. Particularly, the in vitro finding that overexpression of C1GALT1 increases the neurite outgrowth of NB cells is consistent with the high expression of C1GALT1 observed in the NB patients with differentiated tumor histology. These results derived from the clinical analysis of NB patients to phenotypic cellular assays provide new insights that C1GALT1 is considered to exert antitumor effects in NB. Our data provided evidence for the existence of GalNAc-type O-glycans on TrkA and altered O-glycan structures by C1GALT1 can regulate TrkA signaling in NB. This is the first study to demonstrate the effect of O-glycosylation in TrkA signaling in NB. Results of this study shed light on the novel prognostic role of C1GALT1 in NB, in contrast with adult cancers, and provide new information of C1GALT1 and TrkA on the pathogenesis of NB, which could further help to develop new therapeutic strategies for NB in the future.

## Supplementary information

Supplementary Table 1 and Figures

## Data Availability

The datasets used and/or analyzed during the current study are available from the corresponding author on reasonable request.
