# Supplementary material for: C1GALT1 expression predicts a favorable prognosis and suppresses malignant phenotypes via TrkA signaling in neuroblastoma
Source: Oncogenesis. 2022 Feb 15;11(1):8. doi: 10.1038/s41389-022-00383-w (PMC8847342; doi:10.1038/s41389-022-00383-w)
Supplement: Supplementary file 1 — Supplementary Table 1 and Figures [file 41389_2022_383_MOESM1_ESM.docx]

**SF Table 1. Statistical analysis of immunohistochemistry (IHC) indicates that TrkA expression positively correlates with C1GALT1 in neuroblastoma.**

| Neuroblastoma (n = 46) | | | | |
| --- | --- | --- | --- | --- |
| Characteristics | C1GALT1 intensity (No. of patients) | | | *p* value |
|  | Low | High | Total |  |
| TrkA |  |  |  | 0.001** |
| Low | 27 | 8 | 35 |  |
| High | 2 | 9 | 11 |  |
| Total | 29 | 17 | 46 |  |


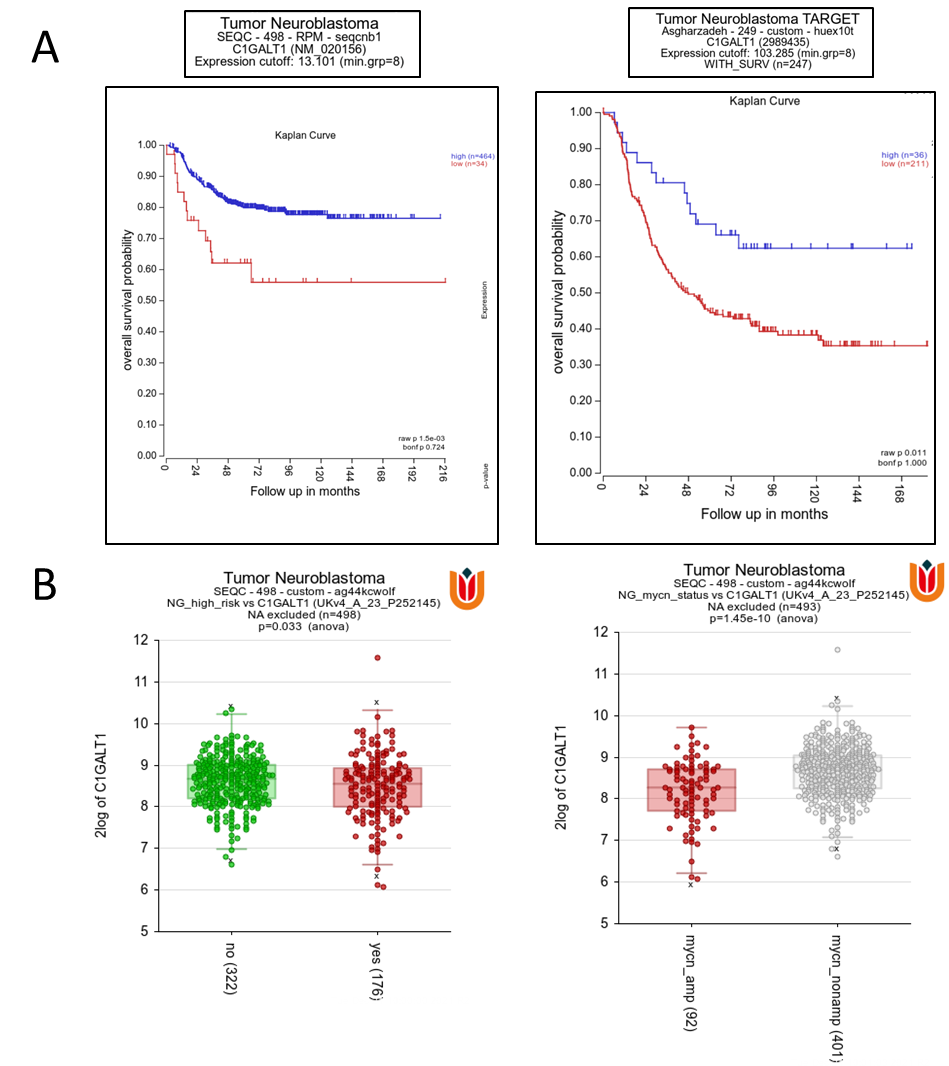


**SF Figure 1.** The analysis of *C1GALT1* mRNA levels in independent cohort studies. (A) Kaplan–Meier curves are shown for overall survival in neuroblastoma patients of SEQC-498 dataset whose tumors expressed low (n = 34) or high (n = 464) levels of *C1GALT1* and Asgharzadeh-249 dataset whose tumors expressed low (n = 211) or high (n = 36) levels of *C1GALT1*. *C1GALT1* high expression in neuroblastoma predicts better survival outcomes compared with *C1GALT1* low expression in these two datasets (*p* = 1.5e-03 and *p* = 0.011, respectively). Scan modus was used for cutoff determination. The R2 Genomics Analysis and Visualization Platform (http://r2.amc.nl) was used to obtain the data. (B) Clinical significance of *C1GALT1* expression. High expression levels of *C1GALT1* were significantly associated with non-high-risk neuroblastoma patients (the left panel, non-high-risk = 322 and high-risk = 176, *p* = 0.033) and MYCN non-amplified patients (the right panel, MYCN amp. = 92 and MYCN non-amp. = 401, *p* = 1.45e-10).


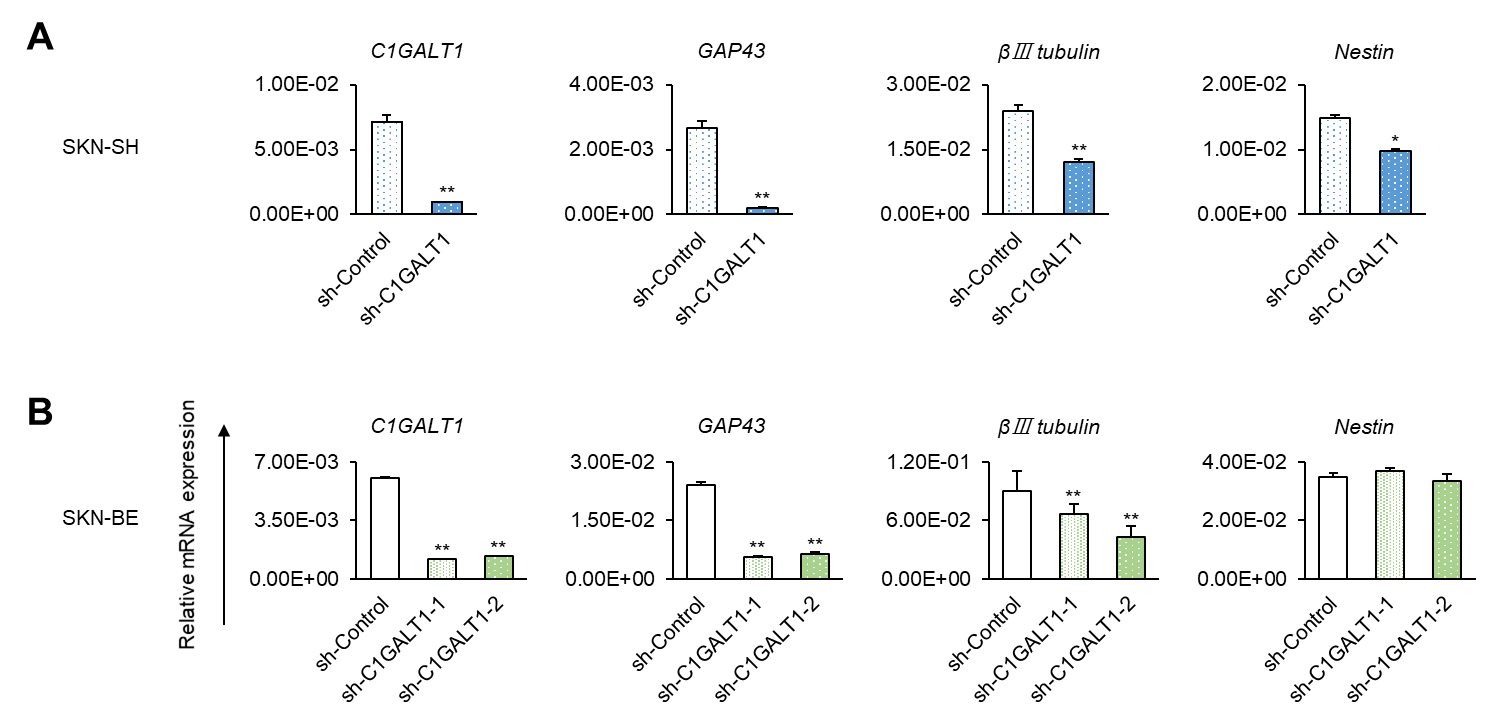


**SF Figure 2.** **C1GALT1 knockdown inhibits mRNA expression of differentiation markers in NB cells.** (A) Data from real-time RT-PCR indicated that C1GALT1 knockdown decreased expression of differentiation markers including GAP43, βⅢ tubulin, and Nestin in SKN-SH cells. (B) Data from real-time RT-PCR indicated that C1GALT1 knockdown decreased expression of differentiation markers including GAP43 and βⅢ tubulin in SKN-BE cells. Data are presented as mean (n = 3) ± SD. **p* < 0.05; ***p* < 0.01.


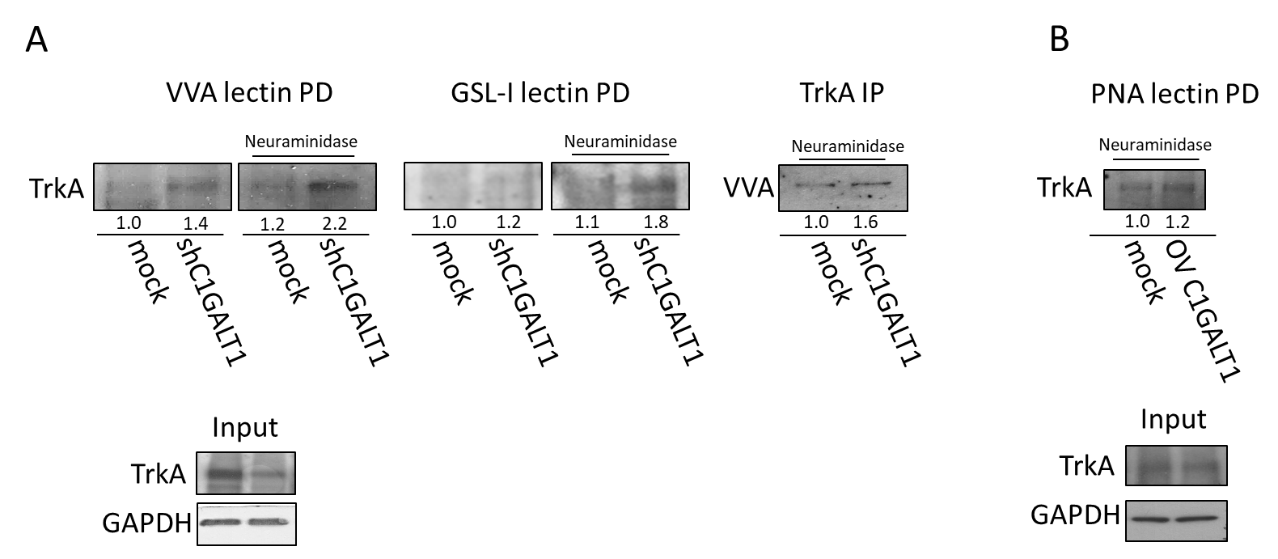


**SF Figure 3.** The glycosylation patterns of TrkA by modifying the C1GALT1 expression. (A) Total lysates treated with or without neuraminidase were pulled down (PD) using VVA, GSL-I agarose beads, or TrkA- protein A sepharose and then Western blotted with a TrkA antibody or VVA lectins. The input of TrkA and GAPDH were shown in the lower panel. (B) Total lysates treated with neuraminidase were pulled down (PD) using PNA agarose beads and then immunoblotted with a TrkA antibody. The input of TrkA and GAPDH were shown in the lower panel.


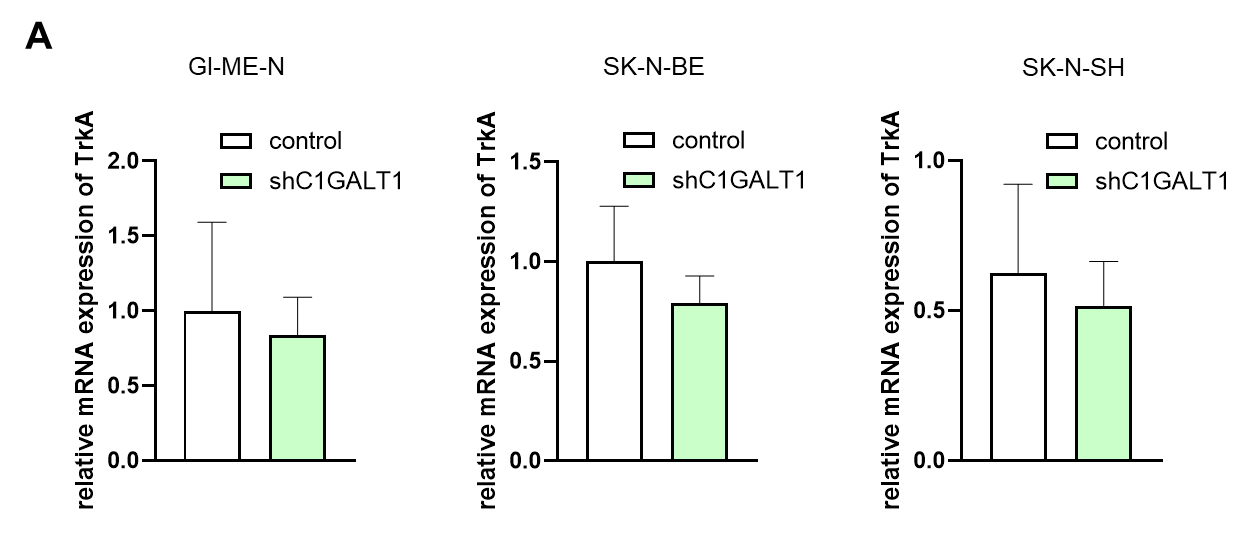


**SF Figure 4.** **C1GALT1 knockdown in NB cells revealed no significant changes in TrkA mRNA levels.** (A) Data from real-time RT-PCR indicated that the mRNA expression levels of TrkA in neuroblastoma cells. β-actin has been used as an internal control. Data are presented as mean (n = 3) ± SD.
